# Effects on gene expression and behavior of untagged short tandem repeats: the case of *arginine vasopressin receptor 1a (AVPR1a)* and externalizing behaviors

**DOI:** 10.1038/s41398-018-0120-z

**Published:** 2018-03-27

**Authors:** Clare C Landefeld, Colin A Hodgkinson, Primavera A Spagnolo, Cheryl A Marietta, Pei-Hong Shen, Hui Sun, Zhifeng Zhou, Barbara K Lipska, David Goldman

**Affiliations:** 1 Cleveland Clinic Lerner College of Medicine at Case Western Reserve University, Cleveland, OH 44195 USA; 2 Laboratory of Neurogenetics, National Institute on Alcohol Abuse and Alcoholism, National Institutes of Health, Rockville, MD 20852 USA; 3 Office of the Clinical Director, National Institute on Alcohol Abuse and Alcoholism, National Institutes of Health, Bethesda, MD 20852 USA; 4 Human Brain Collection Core, National Institutes of Mental Health, National Institutes of Health, Bethesda, MD 20814 USA

## Abstract

Genome-wide association studies (GWAS) of complex, heritable, behavioral phenotypes have yielded an incomplete accounting of the genetic influences. The identified loci explain only a portion of the observed heritability, and few of the loci have been shown to be functional. It is clear that current GWAS techniques overlook key components of phenotypically relevant genetic variation, either because of sample size, as is frequently asserted, or because of methodology. Here we use *arginine vasopressin receptor 1a* (*AVPR1a*) as an in-depth model of a methodologic limitation of GWAS: the functional genetic variation (in the form of short tandem repeats) of this key gene involved in affiliative behavior cannot be captured by current GWAS methodologies. Importantly, we find evidence of differential allele expression, twofold or more, in at least a third of human brain samples heterozygous for a reporter SNP in the *AVPR1a* transcript. We also show that this functional effect and a downstream phenotype, externalizing behavior, are predicted by *AVPR1a* STRs but not SNPs.

## Introduction

Behavioral traits are complex phenotypes with many causative genetic loci. Genome-wide association studies (GWAS) reliant on common single-nucleotide polymorphisms (SNPs) have implicated loci that partially explain the observed heritability of these traits, but have identified few functional loci^1^. The majority of SNPs associated to psychiatric diseases via GWAS are intergenic or intronic and, if functional, would thus be presumed to affect gene expression^2^. However, the variants identified by GWAS may account only for 60% of the observed heritability of cis effects on gene expression^3^. These observations raise the possibility that many of the functional loci influencing risk of psychiatric diseases may be structural variants with cis-acting functional alleles that are not captured by common SNPs. Structural variants include large and small indels, both of which are highly abundant, and that have justifiably attracted recent attention^4^, but also structural elements involving a variable number of copies of sequence motifs.

Short tandem repeats (STRs) and variable number tandem repeat (VNTR) polymorphisms are abundant in the human genome and may alter gene expression or function. STRs, usually defined as tandem repeats of 1–6 bp sequences, and VNTRs, which can be considered tandem repeats of sequences longer than 6 bp, are multi-allelic and hypermutable, the latter due to both strand slippage and unequal crossover during DNA replication and meiosis^5^. As a result, STRs and VNTRs have higher variability and mutability relative to SNPs, and their effects are therefore liable to escape studies based on SNP arrays.

Although individual STR alleles may be evolutionarily transient, STR loci are often conserved across species, which is consistent with functional conservation. Previous studies have demonstrated a variety of functional effects including modulation of RNA splicing^6^ and transcription-factor binding^7^ as well as alteration of tertiary DNA structures^8^. Gymrek et al.^9^ recently underscored the downstream effects of these mechanisms, and suggested that these effects are often not captured by adjacent SNPs, in their genome-wide study of STR effects on gene expression. Despite this strong evidence supporting a functional role of STRs, these polymorphic variants remain understudied relative to other forms of genetic variation due to technically tedious gold-standard genotyping methodologies, and the limitations of next-generation sequencing in genotyping repetitive elements^10^. We used the vasopressin receptor 1a (*AVPR1a*) gene and a behavioral domain, externalization, to which vasopressin has been functionally implicated, as a specific model to examine the functionality of STRs and the ability of neighboring SNPs to capture their effect.

**Fig. 1:**
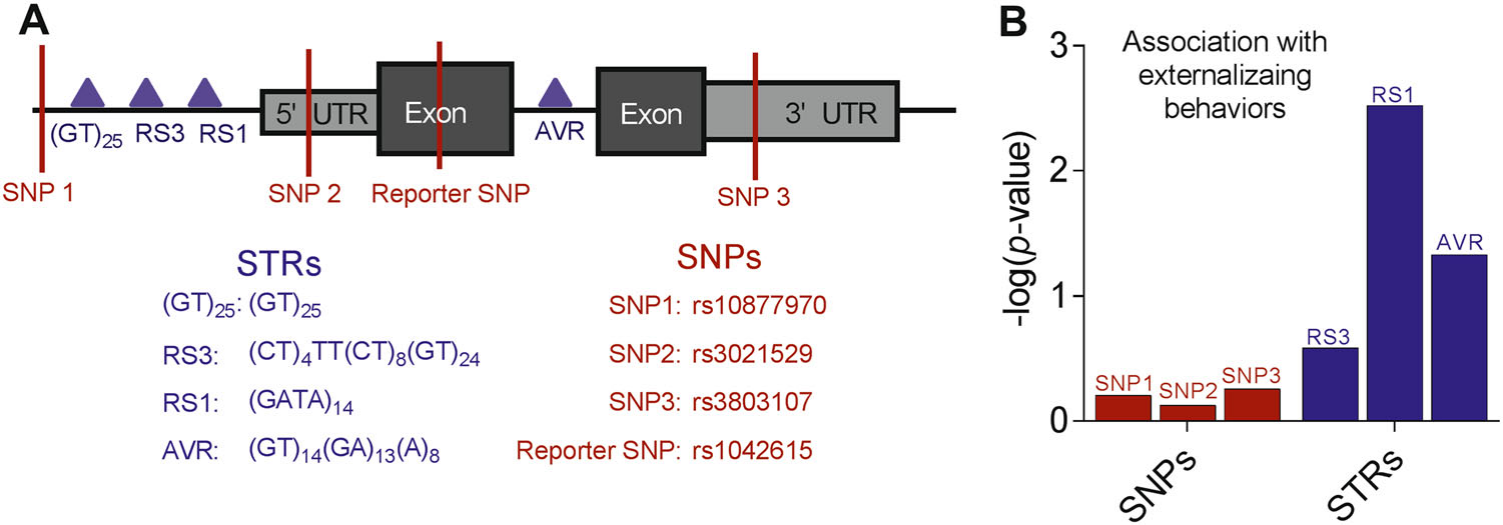
*AVPR1a* STRs and SNPs and their association with Externalizing Behavior. **a** Schematic of *AVPR1a* gene with locations of four genotyped STRs (triangles) and four genotyped SNPs (lines). (GT)_25_ was not genotyped in our study. One of the SNPs is the reporter SNP, which is used as the “barcode” for DAE experiments. **b** Locus-specific global *p* values (as –log) for association with externalizing behavior from logistic regression models controlling for gender. See Table 1 for details of STR models. SNP *p* values are 0.617, 0.745, 0.550, respectively

Vasopressin is a multifunctional neuropeptide whose many effects are partly modulated by the varied distribution of its three distinct receptors. STR polymorphisms in the flanking region of the *AVPR1a* gene (Fig. 1a) are associated with a number of social behaviors in animals and humans including pair-bonding^11,12^ and altruism^13^ as well as diseases such as autism^14^. Preliminary evidence in rodents and humans has demonstrated that these flanking STRs alter behavioral phenotypes by modulating the expression of AVPR1a in various brain tissues^11,13^. Previous work has identified “risk” alleles at three of the four STR loci found in the *AVPR1* gene region: RS1^15,16^, AVR^14^, and RS3^12^.

Externalizing behaviors including impulsivity and aggression show moderate to high heritability^17,18^, but are potentially influenced by many genes. GWAS have explained relatively little of the variance in externalizing behaviors such as ADHD^19^, addictions^20–22^, and suicidality^23^. The vasopressin pathway, and AVPR1a specifically, has been linked with aggression in animal models^24,25^, but the role of *AVPR1a* in externalizing behaviors in humans remains unclear. We hypothesized that variation in the *AVPR1a* STRs drives variation in its expression and has downstream effects on externalizing behaviors, and this effect can be captured by direct genotyping and highly sensitive differential allele expression (DAE). We used postmortem hippocampal samples to assess the effects of *AVPR1a* STRs and SNPS on gene expression, and then tested these loci’s association with externalizing behavior in a cohort of Finnish Caucasians that includes both subjects with severe externalizing behaviors and controls free of psychiatric disease.

## Methods

### Subjects

The human research subjects and postmortem brain samples, their *AVPR1a* genotyping, *AVPR1a* DAE analysis and association to externalizing behavior are summarized in Supplementary Table 1.

#### Finnish Caucasians

The Finnish Caucasian cohort (*n* = 661) consisted of cases with severe externalizing behaviors (*n* = 264) and controls (*n* = 397). Cases were diagnosed with antisocial personality disorder, borderline personality disorder, or intermittent explosive disorder by psychiatrists administering the structured interview for DSM-III-R during forensic psychiatric evaluation after having committed violent crimes or arson. Aggression and impulsivity are common to these diagnoses, and there is evidence that they share genetic components^26^. Controls had no DSM-III-R axis I or II diagnoses. The sample was primarily male (84%). Further demographic information can be found in Supplementary Table 2. Informed consent approved by Institutional Review Boards (IRBs) at the National Institute on Alcoholism and Alcohol Abuse (NIAAA), National Institute of Mental Health (NIMH), University of Helsinki, and the University of Helsinki Central Hospital was obtained from all subjects. Further details about the collection and characteristics of this sample have been previously published^27^.

Within our sample, 98 subjects represented 42 small (*N* = 2) to moderate size (*N* = 12) families; an additional 47 subjects in these families were genotyped, although phenotype information was unavailable on these subjects. Mendelian transmission in the pedigrees was used to validate the accuracy of STR genotype calls (see below). To assess the degree of relatedness within the sample, we used LOKI 2.4.5, a linkage analysis package that uses Markov chain Monte Carlo to calculate a kinship coefficient for each subject–subject pair^28^. The average percentage of shared genetic identity between any two subjects was calculated to be 0.1%, which is less than the degree of relationship between third cousins. We therefore treated the subjects as unrelated, the multi-allelic nature of the STR loci being a limiting factor in using randomization programs such as pLINK (http://zzz.bwh.harvard.edu/plink/)^29^.

#### Postmortem hippocampal samples

Postmortem hippocampal RNA was provided by the Human Brain Collection Core (HBCC), NIMH (*n* = 23), and the NIH Neurobiobank (*n* = 9). Full methods for the collection, preparation, and characterization of the HBCC^30^ and Neurobiobank^31^ samples are detailed in previous work. For the HBCC, informed consent was obtained from the legal next of kin according to the National Institutes of Health Institutional Review Board and ethical guidelines under NIMH protocol (90-M-0142). Subjects were of Caucasian (*n* = 24), African (*n* = 5), Hispanic (*n* = 2), and Asian (*n* = 1) descent and several had psychiatric diagnoses, which was likely not relevant because expression analysis was performed using a DAE method (see below) rather than correlation of genotype to expression.

### SNP genotyping

Finnish subjects (*N* = 661, all completed) were genotyped for eight *AVPR1a* SNPs using Illumina GoldenGate genotyping protocols as previously described^32^. None of the SNPs departed from Hardy–Weinberg Equilibrium. Five SNPs had minor allele frequencies (MAFs) <0.05 and were excluded from the association tests to behavior, but were used to determine SNP to STR genotype/genotype correlations. The 23 HBCC postmortem samples were genotyped for 21 *AVPR1a* SNPs using Illumina Bead Arrays. All 21 of these SNPs were used for association to expression, but not for genotype/genotype correlation because of the small size of the postmortem sample and not for genotype/behavior correlation because of small sample size and lack of the target behavior. The nine postmortem samples from the NIH Neurobiobank were genotyped for the rs1042615 reporter SNP using a Custom Taqman SNP Genotyping Assay (see Supplementary Table 3 for primer and reporter sequences).

### Haplotype construction

*AVPR1a* linkage disequilibrium blocks in the 23 HBCC postmortem samples and 311 of the unrelated Finnish Caucasians were determined using Haploview (https://www.broadinstitute.org/haploview)^33^. Maximum-likelihood estimates of haplotypes were determined using PHASE v2.1 (http://stephenslab.uchicago.edu/software.html#phase)^34,35^. Haplotype clusters were constructed using HapCluster, a cladistic clustering program^36^.

### STR genotyping

Finnsish samples (*n*=363) and the 32 postmortem samples were genotyped for the following *AVPR1a* STRs: RS1, RS3, and AVR. These STRs were PCR-amplified in all subjects using the primers in Supplementary Table 4.

Genotypes were resolved by size using an ABI 3730 Capillary Sequencer and GeneMapper Software v4.0. The accuracy of genotype calls was validated by the construction of three STR locus haplotypes within the Finnish families, and the assessment of Mendelian transmission (mismatch rates for RS1, AVR, and RS3 were 0.003, 0, and 0.02 respectively, Supplementary Table 5). Mutation rates of STRs have been estimated to range from 2.73 × 10^−4^ for dinucleotide repeats and 10.0 × 10^−4^ for tetranucleotide repeats^37^. Importantly, not all genotyping errors result in mismatch, although mismatch is more likely to be detected for multi-allelic STR loci. Conversely, most mismatches would represent one genotyping error, or de novo mutation, rather than two given that frequency of Mendelian mismatch is low, as was observed here. Our STR mismatch rates can therefore be accounted for by a number of factors: expected germline mutations, somatic mutations, and genotyping artifacts, but are representative of a high degree of genotyping fidelity.

### Differential allele expression

**Fig. 2:**
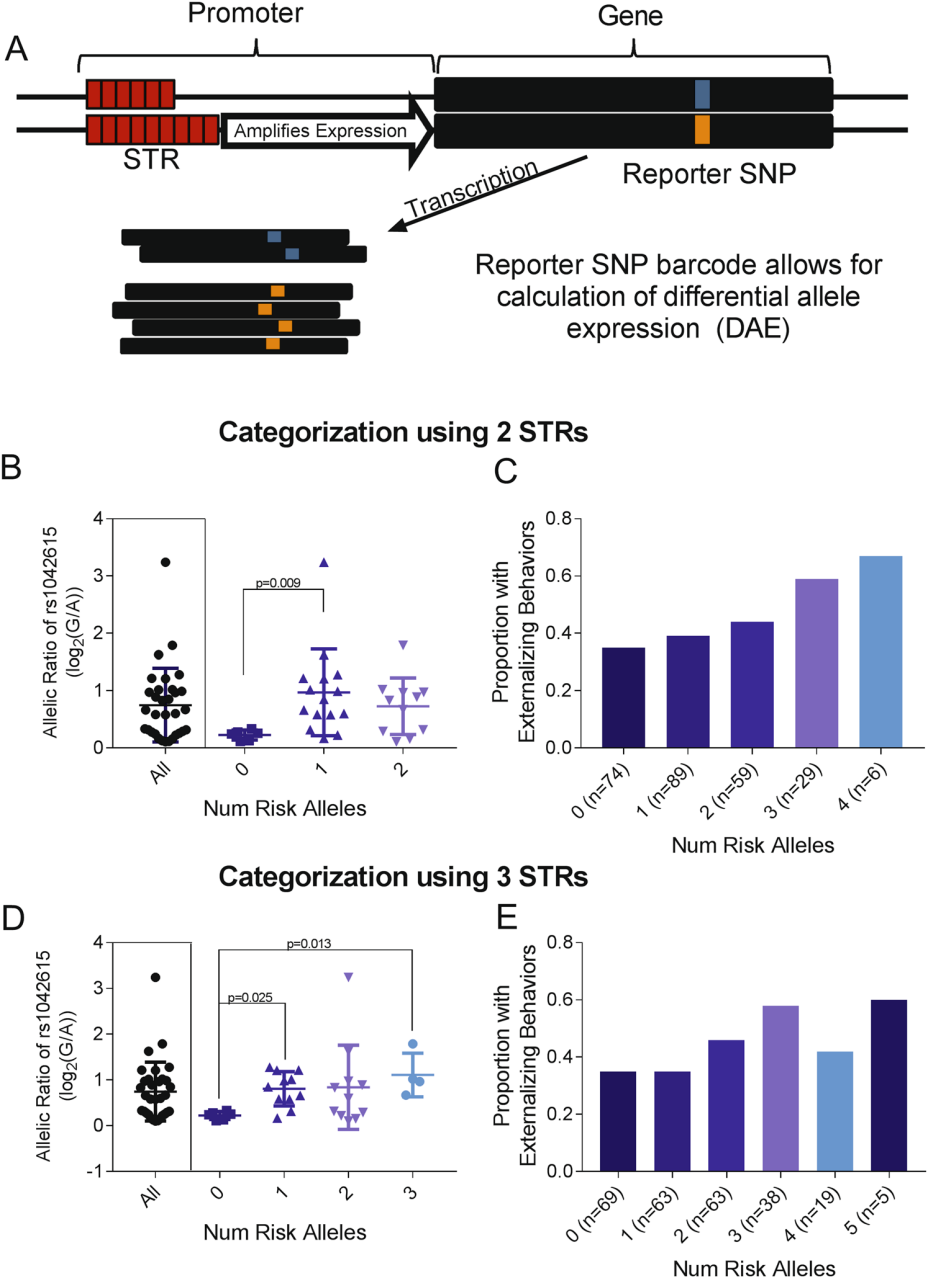
Differential allele expression (DAE) at *AVPR1a* in postmortem hippocampal samples. **a** Schematic of hypothesized cis-effect of one STR on gene expression (increase in expression represented by white arrow). This effect can be captured in subjects heterozygous for a reporter SNP. STRs were considered together in order to capture an additive effect. Two-locus categorization (defined by RS1 and AVR risk alleles) is associated with (**b**) DAE (*p* = 0.017) and (**c**) externalizing behaviors in a dose-dependent manner (*p* = 0.009). Three-locus categorization (defined by RS1, AVR, and RS3 risk alleles) is associated with (**d**) DAE (*p* = 0.018), but does not improve the effect on (**e**) externalizing behaviors (*p* = 0.025). Each dot represents one subject

To determine if the *AVPR1a* STR is a cis-acting eQTL, the expression of the two alleles for a reporter SNP (rs1042615) in the *AVPR1a* gene was compared in heterozygous subjects (Fig. 2a). The reporter SNP was selected on the basis that it was exonic with an allele frequency approaching 0.5, thereby ensuring that multiple heterozygous samples would be available for analysis. The messenger RNA level for each allele was quantified by real-time PCR on the postmortem hippocampal RNA, using a Custom Taqman SNP Genotyping Assay (see Supplementary Table 3 for primer and reporter sequences). Real-Time PCR was also performed on genomic DNA, which was used to normalize the DAE. Total RNA (1 μg) was reverse-transcribed using the Cloned AMV First-Strand Synthesis Kit (Invitrogen). The complementary DNA (cDNA) was quantified on a QuantStudio 7 Flex Real-Time PCR System in a 10 μL qRT-PCR reaction: 2 μL cDNA, 5 μL Amplitaq Gold 360 PCR MasterMix, 0.25 μL custom primer/probe assay, 2.75 μL water. The reaction conditions were as follows: 10 min at 95 °C, and 45 cycles of: 15 s at 95 °C and 1 min at 60 °C. Each sample was analyzed in triplicate. The average difference in Ct (threshold cycle) between the two alleles for cDNA samples was normalized against the average difference in Ct between the two alleles in genomic DNA samples.

### Statistical analysis

STR allele, SNP, and SNP haplotype effects on DAE were tested using nonparametric rank-order statistics (Kruskal–Wallis test for more than two groups, Mann–Whitney test for less than or equal to two groups). STR risk alleles were combined into two-locus and three-locus scores, where each subject could have 0 to 2, or 0 to 3 risk alleles. SNPs with MAF <0.05 were not tested. For the two-locus and three-locus categorization analysis, each group was compared to the group of score “0” with the Dunn’s multiple correction test. All STR, locus score, and SNP effects on externalizing behaviors in the Finnish cohort were tested in logistic regression models controlling for gender. The locus score was treated as a continuous variable and STRs were treated as categorical variables with allele groups as individual levels. Some STR alleles were grouped together by size due to low representation (i.e., AVR was dichotomized into “short” vs. “long”).

## Results

Both the Finnish Caucasians and the postmortem hippocampal samples were genotyped for three *AVPR1a* STRs: RS1, RS3, and AVR (Fig. 1a). The distributions of the STRs in the Finnish cohort was similar to previous reports^14,16,38^ and none deviated from Hardy–Weinberg equilibrium (Table 1).

**Table 1:** *AVPR1a* RS1, AVR, RS3 allele frequencies and association with externalizing behaviors in logistic regression models controlling for gender

Allele (bp)	Allele frequency (AF)	AF in cases	AF in controls	*p* value
RS1** (*n* = 311)				0.003
306	0.173	0.228	0.136	
310	0.368	0.330	0.394	
314	0.206	0.173	0.228	
318	0.106	0.106	0.106	
322	0.109	0.114	0.106	
326	0.002	0.000	0.003	
330	0.035	0.047	0.027	
AVR^*^ (*n* = *330*)				0.047
208	0.029	0.031	0.028	
210	0.108	0.108	0.108	
212	0.315	0.358	0.286	
214	0.479	0.438	0.505	
216	0.030	0.031	0.030	
218	0.040	0.035	0.043	
*RS3*^NS^ (*n* = *319*)				0.260
250	0.009	0.011	0.008	
252	0.002	0	0.003	
256	0.002	0	0.003	
258	0.033	0.042	0.026	
260	0.091	0.095	0.087	
262	0.214	0.187	0.233	
264	0.302	0.321	0.288	
266	0.075	0.046	0.095	
268	0.155	0.164	0.148	
270	0.014	0.019	0.011	
272	0.009	0.011	0.008	
274	0.083	0.095	0.074	
276	0.006	0.007	0.005	
278	0.005	0	0.008	
280	0.002	0	0.003	

Some alleles are grouped together in the regression analysis because of their rarity. Locus-specific global *p* values obtained by Wald effect tests with moderating effect of gender on externalizing behavior are shown

DAE was conducted on 32 postmortem hippocampal samples (Fig. 2a). The reporter locus rs1042615 showed DAE of two or more-fold in approximately one-third of the 32 brain samples tested, indicating the presence of a cis-acting locus altering AVPR1a expression. The extent of differential allelic expression observed was variable but went as high as 10-fold. The valence (preferred allele) of the differential expression was random indicating that the reporter locus was not itself driving DAE nor was the reporter SNP in strong linkage disequilibrium with the cis-acting functional locus. The STR allele showing the greatest effect on DAE was identified for each *AVPR1a* locus, and these were the shortest RS1 allele (306 bp), the short AVR alleles (208–212 bp), and the RS3 268 bp allele (Supplementary Figure 1). Taken individually, these three *AVPR1a* loci did not strongly predict expression (Supplementary Figure 1), but taken together, the number of risk alleles present in a subject predicted DAE (two-locus score *p* = 0.017, Fig. 2b, three-locus score *p* = 0.018, Fig. 2d).

**Fig. 3:**
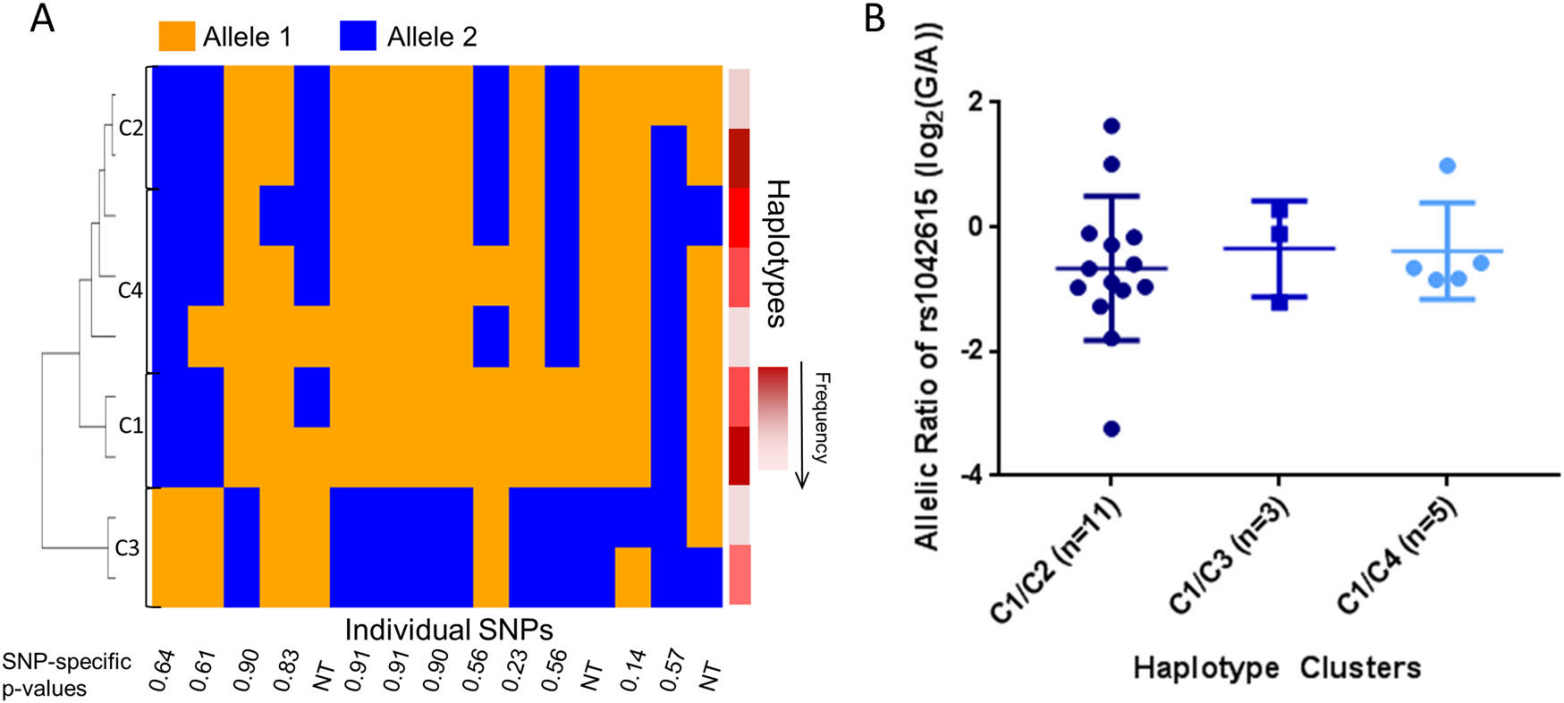
*AVPR1a* SNPs and SNP haplotypes do not predict *AVPR1a* DAE. **a** *AVPR1a* SNPs (as represented by columns) were genotyped and tested for association with DAE (SNP-specific *p* values are below each column). The SNPs were also considered as haplotypes (indicated on the right *Y* axis with haplotype frequency increasing from light to dark red), which were grouped into related clusters based on their phylogeny. Three major *AVPR1a* SNP haplotype clades exist in our DAE sample although in order to compare the effects of the other clusters on DAE, one cluster was split into C2 and C4. **b** The majority of samples had the clade indicated by C1, so the effects of the other clusters could be compared. The SNP haplotype clusters were not associated with DAE (*p* = 0.950)

Individual *AVPR1a* SNPs or SNP haplotypes did not predict *AVPR1a* DAE (Fig. 3). The haplotypes constructed from the 21 *AVPR1a* SNPs in the 23 HBCC postmortem samples coalesced into three clusters, although in order to have multiple groups for our DAE analysis, we split one cluster into two (Fig. 3a). The majority (*n* = 22) of the subjects had cluster 1 (C1), so the effects of the other three clusters (C2, C3, and C4) relative to C1 were determined. None of the individual SNP or haplotype clusters was associated with DAE (Fig. 3b).

Association of the three *AVPR1a* STR loci (using all alleles at these loci) and three *AVPR1a* SNPs to externalizing behavior was tested in logistic regression models controlling for gender. Two of the three STRs showed the strongest associations (Fig. 1b, Table 1: RS1 *p* = 0.003, AVR *p* = 0.047, RS3 *p* = 0.260), and the SNPs showed no association. The RS1 306 bp allele and the short AVR alleles (which in both cases predicted DAE) were associated with an increased prevalence of externalizing behaviors. The RS1 306 allele showed a strong dose-dependent association with externalizing behavior (*p* = 0.005, homozygous OR = 5.12), and AVR short alleles showed a trend toward a dose-dependent association, although ultimately not significant (*p* = 0.110) (Table 2, Supplementary Figure 2). Interestingly, the RS3 locus, which most strongly predicted DAE, was not significantly associated with externalizing behaviors (Table 1).

**Table 2:** Allele-dose-dependent association of the RS1 306 allele and AVR short allele with externalizing behaviors in a logistic regression model controlling for gender

Predictors	Odds ratio (95% CI)	*p* value
*RS1 model*		
Gender (M)	14.6 (4.1, 93.7)	<0.001
RS1 306 genotype		0.005
0, Non-carrier	1.00	
1, Heterozygous	2.04 (1.20, 3.50)	
2, Homozygous	5.12 (1.38, 24.2)	
*AVR model*		
Gender (M)	6.55 (2.51, 22.5)	<0.001
AVR short genotype		0.110
0, Non-carrier		
1, Heterozygous		
2, Homozygous		

The two-locus STR (including RS1 and AVR) and three-locus STR (including all three STRs) combinations predicting DAE were used for behavioral association analysis. The two-locus score predicted presence of externalizing behaviors in a dose-dependent manner (*p* = 0.009, Fig. 2c). The three-locus score also predicted externalizing behaviors, although the relationship was not dose-dependent and the addition of the RS3 locus to the model did not improve prediction (*p* = 0.025, Fig. 2e).

The STRs did not demonstrate strong linkage disequilibrium with SNPs or with each other. STR genotypes could not be imputed from the SNP genotypes alone (Supplementary Figure 3a). When both the STR and SNP genotypes are known, less than one-third of STR-SNP haplotypes can be imputed with high certainty (>0.95) (Supplementary Figure 3a). Combinations of any two of the STR genotypes and the SNP genotypes were insufficient to predict the third STR (Supplementary Figure 3b).

## Discussion

We sought to explore the limits of current genome-wide association tools to detect behaviorally relevant functional genetic variation by examining one gene that has been strongly implicated in affiliative behavior and that has multiple STR loci in close proximity: *AVPR1a*. We aimed to determine the functional effect of genetic variation in the *AVPR1a* gene, the source of this genetic variation, and whether this functional effect could be captured by SNPs. We have shown that common genetic variation in *AVPR1a* has important functional effects, resulting in changes of expression greater than twofold in at least one-third of samples. We have provided evidence that the *AVPR1a* STRs predict this functional effect as well as downstream behavioral traits (externalizing behavior). Moreover, we demonstrate that this functional and phenotypic effect cannot be captured by *AVPR1a* SNPs.

Across worldwide populations approximately one in two people are heterozygous for the AVPR1a reporter locus we used, and among brain samples heterozygous for this reporter locus, one-third showed DAE, with an effect size between twofold and 10-fold. Perhaps most importantly, the *AVPR1a* SNPs associated with neither DAE nor externalizing behavior, supporting the idea that the genotyped STRs are responsible for the functional effect, or are more capable of predicting this effect given their multi-allelic nature. Moreover, in our study, the SNPs are not in high linkage disequilibrium with the STRs, so this locus would not be identified on a SNP GWAS. Indeed, no GWAS to date has identified *AVPR1a* as a risk locus although our data show that cis-acting functional alleles are frequently present. In these studies, the effect size or the sample size of the study could be too small to capture *AVPR1a*, but we also raise the possibility that the SNP arrays used do not tag the functional *AVPR1a* variation. Interestingly, confirmed cis-acting functional alleles are frequently not represented in GTEX^39,40^ (www.gtexportal.org) and other inventories of cis-eQTLs based on SNP genotype/expression correlations. For example, although there are 189 eQTLs reported for *AVPR1a* with an absolute effect size (slope) of >0.20 in 399 thyroid samples, there is 1 *AVPR1a* eQTL reported for 80–154 samples representing 13 brain regions. Similarly, there are 524 eQTLS reported for *SLC6A4* in the 361 tibial nerve samples, but none reported for the brain samples, despite a well-known cis-acting eQTL at this gene, namely HTTLPR^41^. SNP-based eQTL databases include both false negatives and false positives, due to methodological limitations^42^, but, as shown here and in line with previous reports of low STR-SNP linkage disequilibrium^9^, also because of potential genetic mismatch between the SNPs and the functional loci they are being used to capture.

The effect of the *AVPR1a* STRs on DAE was best captured by a multi-locus approach, implying that at least at this gene there may be more than one functional STR locus, or that the combined information from the STRs better captures the effect of an unknown locus. “Risk” alleles at each of these STRs showed trending associations with DAE and were included in the multi-locus approach. The combined effect of these risk alleles was associated with both DAE and with the phenotype of externalizing behavior. For RS1 and AVR, these risk alleles represent short alleles, and are supported by the literature^14,16^. These two alleles were also associated with externalizing behaviors, RS1 in a dose-dependent manner, and AVR with a dose-dependent trend. Interestingly, for RS3, the most complex and polymorphic of the STRs, the allele that showed the strongest association with DAE has not been previously implicated in the literature. This allele was also not associated with externalizing behavior in our cohort. The DAE effect of RS3 could be inflated by the relatively small number of carriers in our DAE samples. Unsurprisingly, this RS3 allele that showed the strongest association with DAE but was not associated with externalizing behavior did not improve the multi-locus score. Interestingly, the RS1 allele showed the weakest independent association with DAE, but contributed to the multi-locus approach and showed the strongest association with externalizing behavior.

The additive effect of the STR loci is consistent with the weak linkage disequilibrium between the STRs. If the STR alleles were highly correlated, or if one STR locus accounted for all the cis-effect on expression, we would expect one locus to capture the DAE and behavioral effect rather than the additive effect we observe. In line with observations of genetic heterogeneity in other heritable diseases (e.g., cancer of the breast and ovaries, cystic fibrosis), multiple functional polymorphisms and a plethora of uncommon and rare variants that alter function and risk are expected to be detected within the same gene (i.e., *BRCA1*, *CFTR*)^43,44^.

The complexity of the three *AVPR1a* STRs we studied is reflected in their multi-allelic nature, ranging from 6 alleles (AVR) to 15 alleles (RS3). Even with the gold-standard capillary sequencing method of STR genotyping, the nature of repetitive elements translates to significant genotyping noise due to stutter during DNA amplification. Moreover, repetitive elements have proven to be one of the most difficult forms of genetic variation to capture using next-generation sequencing techniques due to the inherent ambiguity in mapping reads of sequence repeats. Even STR-mapping algorithms and targeted-fragmentation techniques are limited by the size of the Illumina reads (typically limiting the size of repeats to <100 bp). Our study is thus strengthened by the presence of nuclear pedigrees in our sample, which we could use to validate genotype calls.

GWAS of complex behavioral traits and psychiatric pathologies have identified many SNPs that contribute to the observed heritability of these phenotypes, but have identified few functional loci. For example, in the 108 genes, including complement C4^45^, implicated in schizophrenia^1^ by the Psychiatric Genomics Consortium, no functional locus has been identified. Furthermore, polygenic risk score (PRS) analyses are based on the premise that hundreds, or even thousands, of loci contribute to the inheritance^1,46,47^, and cross-inheritance^48^ of psychiatric diseases, but in PRS, it is unclear which of the nominally significant SNPs represents a contributing locus based on the statistical criteria by which they were selected. Several of these GWAS and daughter PRS results have been replicated in other samples, but full validation, and the clinical application and extension of these findings via molecular neuroscience, requires the identification of the functional locus. Indeed, effects on behavior are small and difficult to replicate, a challenge that underscores the advantage to our approach of examining the effects of polymorphisms on molecular function, where effects can be stronger. Various factors, including etiologic heterogeneity of phenotypes^49^ can account for the “missing heritability” of psychiatric diseases in genomic studies, but here the focus is on genotype, rather than phenotype. An estimated one- to two-thirds of functional genetic variants can be tagged by common SNPs and structural variants such as STRs may account for many of the untagged variants^50^. Recent reports highlighting the impact of structural variants on gene expression^4^ reinforce the idea that this largely ignored category of genetic variation explains a piece of the missing heritability, at least for gene expression, and thereby for traits altered by variable expression of genes.

Several STR and VNTR polymorphisms of neurogenetic candidate genes have been shown to be functional^41,51–53^ and linked to both complex behaviors and to intermediate phenotypes for psychiatric diseases. For example, reports have associated these variants with differences in gene expression, brain structure, and ligand-binding or task-evoked metabolic responses^54–56^. It is important to note that while associations of these STR and VNTR loci to complex behavioral phenotypes have remained somewhat controversial, and despite many meta-analyses that would—for some of the locus-phenotype associations—seemingly have settled the issue, there is little doubt that these alleles modulate the in vitro and in vivo expression of the genes. For example, in the case of HTTLPR, the effect of the VNTR on expression of the serotonin transporter gene (*SLC6A4*) is mechanistically understood^41^ and coherent with reduced expression of this transporter in the living^57^ and postmortem^58^ brain, and with consequences for enhanced metabolic responses of brain regions involved in processing emotional stimuli^59^. Among neurologic diseases, Huntington’s Disease, which can initially present clinically as psychosis, as well as Spinocerebellar Atrophy, Friedrich’s Ataxia, and different forms of Fragile X Syndrome are all caused by trinucleotide repeat sequences that are hypermutable in the germline and undergo catastrophic expansion in somatic tissues, in turn altering gene expression and leading to disease. None of these genes was detected by GWAS, or via high-density SNP arrays.

Our study has several important limitations. First, although our DAE results suggest an STR effect on expression, the associative nature of this method means that the functional locus could be in linkage disequilibrium with the STRs. This work has no clinical implications and interventional molecular studies are needed to identify the functional loci driving *AVPR1a* DAE and the downstream phenotype. Second, the number of SNPs genotyped for the Finnish cohort was limited to eight, and only three had sufficient MAF to be tested for association. It is possible that other SNPs are associated with externalizing behavior but were not included in our study. However, *AVPR1a* is relatively small (~11,000 bp) and, although the SNPs in this region do not tag the STRs, they are in high linkage disequilibrium with each other. Information on other SNPs, but not rare SNVs, would be captured by the three SNPs we tested for association to behavior, and the larger number (*n* = 21) we evaluated for effect on *AVPR1a* expression. In addition, given the small allele frequency of some of the STR alleles, we grouped some alleles by size. Although there is evidence that in many cases, STR effect on expression is proportional to length^11,60^, this may not always be the case^61^. In the case of AVR, such a grouping has not previously been reported, however, our designation as short and long alleles was functionally supported given that the short alleles had larger DAE than the longer alleles. Finally, given that the STRs are not in linkage disequilibrium, our DAE methodology relying on a reporter SNP was not sufficient to determine the direction of the gene expression effect. However, given previous reports in the literature of shorter *AVPR1a* repeat alleles being associated with reduced promoter activity and reduced expression^11,13,38^, and because of the role of this receptor in affiliative behavior^11,12^, we believe the associations to externalizing behavior we detect are most likely to be driven by reductions in *AVPR1a* expression.

Here we presented a model that adds to an accumulating body of evidence that non-SNP variants at genes integral in behavior, but poorly captured by SNP arrays, can be functionally important. The solution to these dilemmas would appear to be the direct measurement of cis-eQTLs by methods such as DAE, and the genotyping of STRs.

## Electronic supplementary material

Supplementary Material(PDF 510 kb)
