# Supplementary material for: Effects on gene expression and behavior of untagged short tandem repeats: the case of arginine vasopressin receptor 1a (AVPR1a) and externalizing behaviors
Source: Transl Psychiatry. 2018 Mar 27;8:72. doi: 10.1038/s41398-018-0120-z (PMC5913313; doi:10.1038/s41398-018-0120-z)

SUPPLEMENTARY INFORMATION

Supplementary Table 1. Summary of samples, and the genotyping and analysis performed for each.

Sample		Sample Size	*AVPR1a* SNPs Genotyped	*AVPR1a* STRs Genotyped	Analysis
Finnish Caucasians		661	8	3	• Behavioral Association<br>• Genotype Correlations
Post-Mortem Hippocampal RNA	HBCC	23	21	3	• DAE
	NIH Neurobank	9	1 (DAE Reporter SNP)	3	• DAE

Supplementary Table 2. Demographic information for Finnish Caucasians

	Cases	Controls
N	264	397
Male (%)	254 (96)	304 (77)
Mean Age in years	34.9	40.3

Supplementary Table 3. Primer and reporter sequences for rs1042615 Custom Taqman Genotyping Assay

Assay Name	Primer	Primer Sequence	Reporter	Reporter Name	Dye	Reporter Sequence
ANFVKX2 (*AVPR1a*/ rs1042615)	Forward	GTGCCGCGTGGTGAAG	1	ANFVKX2_M	FAM	CGACGC**G**AACATG
	Reverse	GTCGGCTGTCATGACTACCA	2	ANFVKX2_V	VIC	CGACGC**A**AACATG

Supplementary Table 4. Primers used for STR genotyping.

STR	Primer
RS1	5’-AGGGACTGGTTCTACAATCTGC-3’ (forward)<br>5’-ACCTCTCAAGTTATGTTGGTGG-3’ (reverse)
RS3	5’- TGCTCAAAGGCACACTGTTCTCA-3’ (forward)<br>5’- TTGGGAATCTGGTCAGGTAAGTG-3’ (reverse)
AVR	5’- ATCCCATGTCCGTCTGGACCG-3’ (forward)<br>5’- AGTGTTCCTCCAAGGTGCGAC-3’ (reverse)

Supplementary Table 5. STR Mismatch Rates in Finnish pedigree sample (N= 145)

STR	# of Alleles showing non-Mendelian transmission	Mismatch Rate
RS1	1	0.003
AVR	0	0
RS3	6	0.02

Supplementary Figure 1. Differential allelic expression observed for each of the three *AVPR1α* STR loci. STR risk alleles are not independently strongly associated with DAE for RS1 (p=0.530), AVR (p=0.086), and RS3 (p=0.043) respectively. Each dot represents one subject.

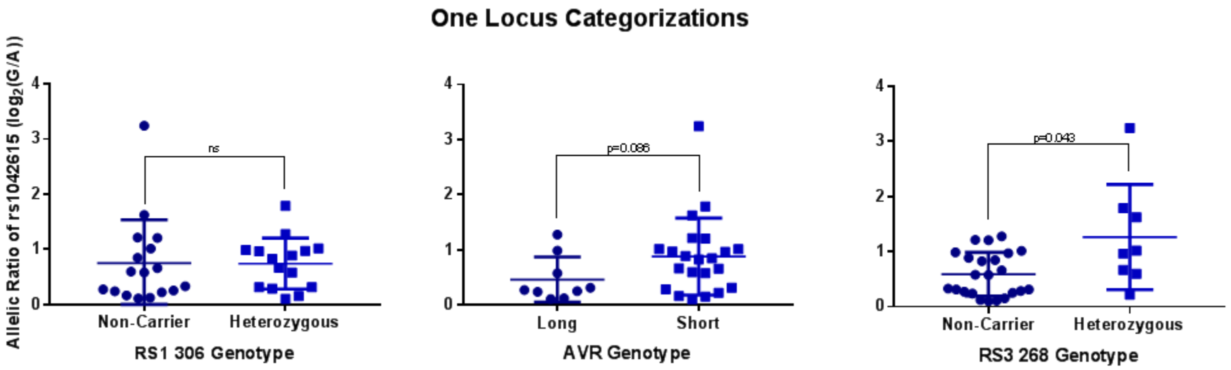

Supplementary Figure 2. Allele-dose-dependent association of the RS1 306 allele and AVR Short allele with externalizing behaviors.

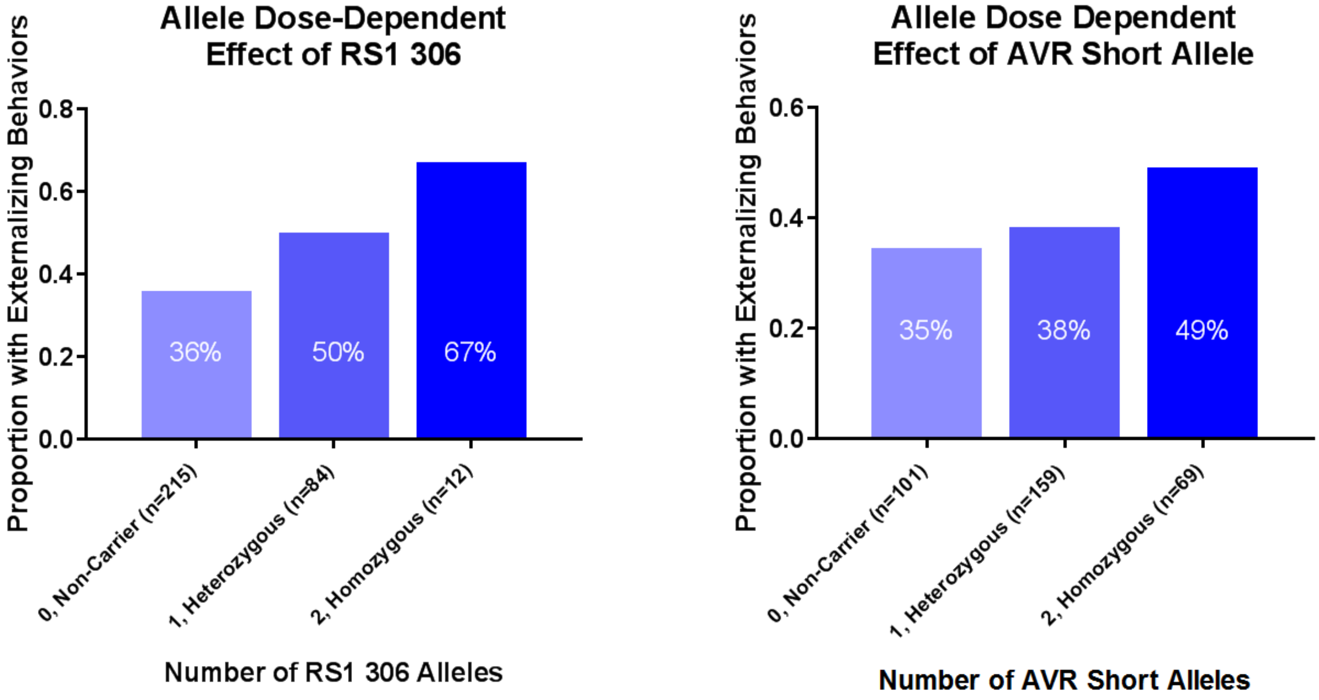

Supplementary Figure 3. STR genotypes and SNP genotypes are not highly correlated. Haplotype reconstruction was attempted with the unphased genotypes of the 311 unrelated Finnish Caucasians and the maximum likelihood of haplotype estimates is shown. **a**. SNP genotypes do not predict STR genotypes. Median maximum likelihood for all subjects ("Total") was 0.34. Of these subjects that had genotyped STRs and SNPs, the maximum likelihood was higher ("With STRs": median = 0.71) relative to those for whom only the SNP genotypes were known ("Without STRs": median = 0.042). **b**. STR genotypes do not predict each other. AVR, RS3, and SNP genotypes cannot predict RS1 genotypes (median maximum likelihood = 0.25), RS1, RS3, and SNP genotypes cannot predict AVR genotypes (median maximum likelihood = 0.42), and RS1, AVR, and SNP genotypes cannot predict RS3 genotypes (median maximum likelihood = 0.31).

a b

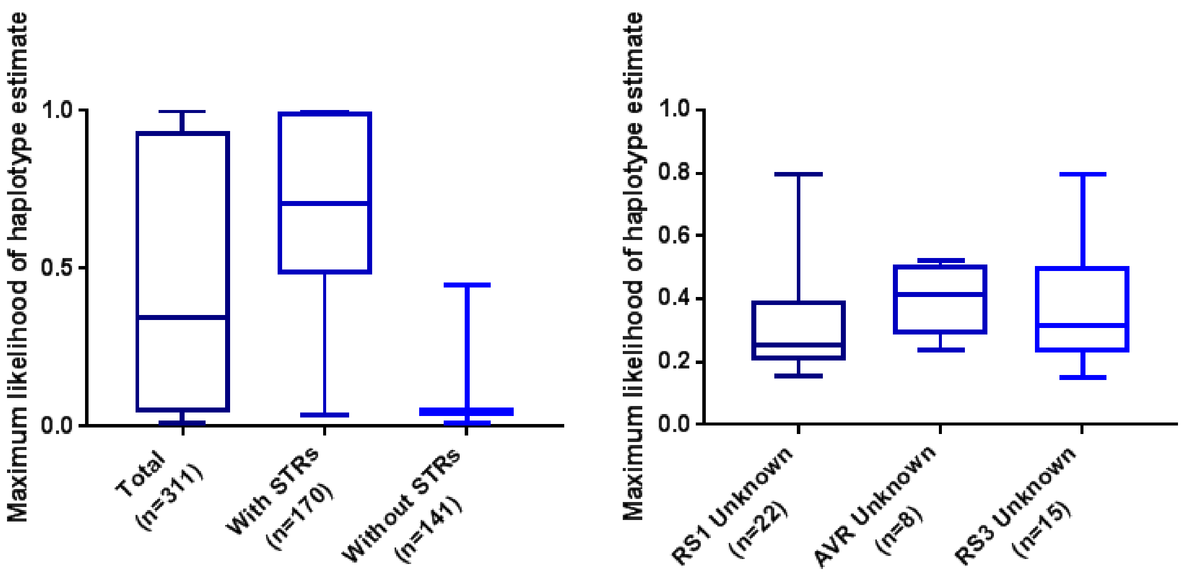

Supplement: Supplementary file 1 — Supplementary Material(PDF 510 kb) [file 41398_2018_120_MOESM1_ESM.pdf]
